# Patient Challenges and Needs in Comprehending Laboratory Test Results: Mixed Methods Study

**DOI:** 10.2196/18725

**Published:** 2020-12-07

**Authors:** Zhan Zhang, Daniel Citardi, Aiwen Xing, Xiao Luo, Yu Lu, Zhe He

**Affiliations:** 1 School of Computer Science and Information Systems Pace University New York, NY United States; 2 Department of Statistics Florida State University Tallahassee, FL United States; 3 School of Engineering and Technology Indiana University-Purdue University Indianapolis Indianapolis, IN United States; 4 School of Information Florida State University Tallahassee, FL United States

**Keywords:** consumer health information, health information technology, patient portal, clinical laboratory test

## Abstract

**Background:**

Patients are increasingly able to access their laboratory test results via patient portals. However, merely providing access does not guarantee comprehension. Patients could experience confusion when reviewing their test results.

**Objective:**

The aim of this study is to examine the challenges and needs of patients when comprehending laboratory test results.

**Methods:**

We conducted a web-based survey with 203 participants and a set of semistructured interviews with 13 participants. We assessed patients’ perceived challenges and needs (both informational and technological needs) when they attempted to comprehend test results, factors associated with patients’ perceptions, and strategies for improving the design of patient portals to communicate laboratory test results more effectively. Descriptive and correlation analysis and thematic analysis were used to analyze the survey and interview data, respectively.

**Results:**

Patients face a variety of challenges and confusion when reviewing laboratory test results. To better comprehend laboratory results, patients need different types of information, which are grouped into 2 categories—generic information (eg, reference range) and personalized or contextual information (eg, treatment options, prognosis, what to do or ask next). We also found that several intrinsic factors (eg, laboratory result normality, health literacy, and technology proficiency) significantly impact people’s perceptions of using portals to view and interpret laboratory results. The desired enhancements of patient portals include providing timely explanations and educational resources (eg, a health encyclopedia), increasing usability and accessibility, and incorporating artificial intelligence–based technology to provide personalized recommendations.

**Conclusions:**

Patients face significant challenges in interpreting the meaning of laboratory test results. Designers and developers of patient portals should employ user-centered approaches to improve the design of patient portals to present information in a more meaningful way.

## Introduction

### Motivation

Rapid and accurate communication of laboratory test results to patients is critical [1]. Growing evidence suggests that patients are increasingly interested in direct access to their test results, regardless of the indication of the results [2,3]. Policies, guidelines, and initiatives have been implemented to encourage health care organizations to provide patients with easy access to their test results through patient-facing technologies [4,5], such as patient portals, which are often linked to electronic health record systems [6,7]. It is well recognized by the health informatics community that increasing patients’ access to their data can lead to better patient-centered medical care [8], greater patient engagement in medical decision making [3,4,9], and enhanced patient-clinician relationship [10,11]. Despite the potential benefits of such direct access, there are long-standing yet unresolved concerns that many patients may not be able to fully understand their laboratory test results and evaluate the indications for their health [12,13].

Why does merely providing access to laboratory test results not guarantee comprehension? First, laboratory tests are complex. Even medical professionals cannot efficiently and accurately interpret them [14], let alone patients. Second, many patients have limited health literacy and numeracy, thereby making it difficult to interpret the test results and decide corresponding actions (eg, call their doctor for an urgent appointment vs regular follow-up visits) [15,16]. Finally, the current interface of patient portals also poses significant challenges in viewing and comprehending test results [15,17,18]. Many portals only provide test results to patients in a tabular format with standard reference ranges, similar to the format received by clinicians. As such, these portals could be of little use to those who are supposed to benefit from them most [15,19]. Although some visual aids, such as color, have been used in patient portals to help patients identify out-of-range values [20], test results are often presented with unfamiliar abbreviations and units, lacking guidance on whether the results are concerning [21]. Therefore, lay individuals have difficulty identifying meaningful information out of their test results and making informed decisions [22].

Despite the urgent need to address these issues, only a few studies have looked into how to better deliver test results along with the support of people with specific needs [23,24]. There is a lack of in-depth, empirical understanding of patients’ experiences, challenges, and needs related to comprehending laboratory test results via patient portals. To that end, we conducted a mixed methods study to address this research gap by examining the 3 following research questions (RQs):

- RQ1: What are patients’ perceived challenges and needs when they attempt to comprehend test results?
- RQ2: What factors are associated with patients’ perceptions?
- RQ3: How can we improve the design of patient portals to communicate laboratory test results to patients in a more meaningful and understandable manner?

### Related Work

The literature on supporting patients’ comprehension of test results is sparse but growing. A few studies attempted to understand patients’ confusion about their laboratory test results by examining online posts. For example, Reynolds et al [25] examined patient question posts in an online health forum and found that patients asked questions about diagnosis, treatment, laboratory report, risk, and prognosis. In a similar vein, Zhang et al [26] examined question posts in a social question and answer site and found that patients’ confusions primarily centered on understanding the meaning of laboratory values, specific terminology, and the causes of abnormal or inconsistent results. However, one major limitation of these studies is the lack of direct patient input; as indicated by previous work, failing to involve patients in the process of developing patient-facing applications could lead to limited uptake of these systems [27]. Therefore, a comprehensive understanding of patients’ needs is of utmost importance for designing supportive technologies.

A few studies addressed this limitation by eliciting patient opinions and insights through user studies, such as surveys and interviews. For example, Giardina et al [23] examined patient perceptions of receiving test results via online portals and found that most patients did not receive any explanatory information at the time they received the result. They also stated that current online portals are not designed to present test results to patients in a meaningful way. In another study, Reiff et al [28] conducted semistructured interviews with parents of pediatric outpatients to identify the needs of families undergoing chromosomal microarray (CMA) testing. They found that incomplete comprehension of test results and scientific uncertainty were prominent challenges faced by families. Receiving results from nongeneticists and misleading internet searches, among others, was found to contribute to the misunderstanding of CMA testing results. Building upon their work, we conducted a mixed methods study to gain further insight into patients’ challenges and needs when comprehending laboratory test results.

Gaining an in-depth and thorough understanding of patients’ information needs, faced challenges, and preferences when comprehending laboratory test results is vital to inform the design of patient portals and other patient-facing informatics tools [21]. However, only a few studies explored patients’ information needs and challenges when interpreting their test results and how to improve the design of patient portals for better support. For example, Nystrom et al [29] designed and evaluated a patient-centered test result interface that consists of visual ranges of laboratory values, nontechnical descriptions of the test and the result, and links to reputable internet resources where patients could learn more about their result. User testing indicates that these features were perceived as usable because they account for patients’ needs and preferences for understanding laboratory test results. Zikmund-Fisher et al [21] tested the effect of presenting patients with an additional reference point in visual displays of laboratory test results regarding when test results become clinically concerning. They found that providing patients with such evaluative cues can substantially reduce the perceived urgency of out-of-range results that normally do not require immediate action. These studies, however, did not have a user study component; therefore, patients were left out of the system design process. This study contributes to this line of research by examining patients’ needs through holistic user studies and leveraging the findings to suggest design implications for patient portals to promote patients’ comprehension of test results.

### Aim of This Study

The ultimate goal of this study is to understand the design requirements and specific strategies for improving patient portals to communicate laboratory test results to patients in a way that is more meaningful and understandable. The development of these requirements and strategies requires an empirical understanding of patients’ perceived challenges and needs when attempting to comprehend test results.

## Methods

### Design

We used a mixed methods approach [30], combining a web-based survey, and semistructured interviews, to gain an empirical and thorough understanding of patients’ experiences, challenges, and needs in comprehending laboratory test results. The reason for using both survey and interview methodologies is that they are suitable for creative knowledge generation of a multilayered issue [31], such as our research context, which encompasses many interdependent factors (eg, people, technology, data, and knowledge). In addition, these 2 methods are complementary to each other, allowing us to compare and triangulate the findings from each study [32]. For example, the survey study allowed us to broadly understand our research questions, whereas the interview study helped us obtain more in-depth insights. Both methods were used jointly to ensure a mix of both broad and deep understanding of the 3 research questions. The survey (Multimedia Appendix 1) was developed in an iterative manner by the researchers and pilot-tested with a small group of people (n=10) to ensure the clarity, appropriateness, and relevance of the questions. The interviews (Multimedia Appendix 2) were informed by the exploratory survey. This study was approved by the Pace University Institutional Review Board (IRB# 19-08).

### Data Collection

Data collection occurred between July and September 2019. We recruited 203 participants from Amazon Mechanical Turk (MTurk) to participate in our survey. Using MTurk to study patients’ perceptions has been proven reliable and effective [33]. To determine their eligibility, we first asked potential respondents whether they had recently taken any laboratory test and whether they had used patient portals to view the results. If they responded with *yes* to both questions, we asked them to read and electronically sign the informed consent form and complete the survey. Toward the end of the survey, we invited them to participate in a follow-up interview study. Potential participants were instructed to email researchers to arrange a suitable time for the interview.

#### Online Survey

Table 1 shows survey participant characteristics, including an almost equal number of male and female participants (104/203, 51.2% and 98/203, 48.3%, respectively). Most of the participants were White (141/203, 69.5%), aged between 26 and 49 years (129/203, 63.5%), had a bachelor’s or higher degree (134/203, 66.1%), had a full-time job (157/203, 77.3%), and self-reported having above medium health literacy (111/203, 54.7%) and technology proficiency (148/203, 72.9%).

**Table 1:** Characteristics of survey participants (N=203).

Participant characteristics		Participants, n (%)
**Age (years)**		
	18-25	38 (18.7)
	26-49	129 (63.5)
	50-64	32 (15.8)
	65 and older	4 (2.0)
**Gender**		
	Male	104 (51.2)
	Female	98 (48.3)
	Other	1 (0.5)
**Race or ethnicity**		
	Asian or Pacific Islander	9 (4.4)
	African American	34 (16.7)
	Hispanic/Latino	12 (5.9)
	American Indian	4 (2.0)
	White	141 (69.5)
	Other	3 (1.5)
**Education**		
	Doctorate degree	6 (3.0)
	Master’s degree	32 (15.8)
	Bachelor’s degree	96 (47.3)
	Associate degree	28 (13.8)
	High school degree	40 (19.7)
	Other	1 (0.4)
**Employment status**		
	Unemployed	17 (8.4)
	Part time	23 (11.3)
	Full time	157 (77.3)
	Other	6 (3.0)
**Employment industry**		
	Government	16 (7.9)
	Health care	23 (11.3)
	Education	32 (15.8)
	Finance	22 (10.8)
	Information technology	44 (21.7)
	Other	66 (32.5)
**Health literacy**		
	High	44 (21.7)
	Medium-high	67 (33.0)
	Medium	81 (39.9)
	Low-medium	10 (5.0)
	Low	1 (0.4)
**Technology proficiency**		
	High	67 (33.0)
	Medium-high	81 (39.9)
	Medium	52 (25.6)
	Low-medium	2 (1.0)
	Low	1 (0.5)

The survey assessed 3 domain areas: (1) participants’ sociodemographic characteristics, (2) participants’ experiences and information needs about understanding laboratory results, and (3) participants’ perceptions of reviewing results via patient portals. Participants’ characteristics collected included age, gender, race/ethnicity, education, and occupation. As health literacy and technology proficiency may affect patient use of the patient portal to view test results [15,19,34], we also asked them to rate their technology proficiency and health literacy on a scale of 1 to 5 (*1* denotes low literacy, whereas *5* denotes high literacy). Patients’ experiences include whether they understand the results, what actions have been taken to make sense of the test results, if they have confusion regarding the result, and what types of information or advice are needed. Patients’ perceptions of the patient portal include the usability of patient portals, challenges or concerns (if any) with using patient portals, and tailoring the patient portal to their needs.

We directed participants to answer survey questions based on their most recent experience of reviewing laboratory test results in a patient portal. However, respondents may have difficulty recalling such an experience, and thus, we provided a commonly seen laboratory test—the lipid profile (eg, total cholesterol, low-density cholesterol, and high-density cholesterol)—to help participants situate themselves in the context (Table 2). The test results were presented following the format currently implemented in many patient portals, that is, using tables to show test values and units. The reason for choosing this laboratory test is that it is routinely performed among adults for screening and diagnostic purposes; therefore, many adults are likely familiar with the test [35]. The survey took about 10-15 min to complete, and the respondents received US $2 after they completed the survey.

**Table 2:** Lipid profile.

Type	Reference range	Results
HDL^a^ cholesterol	>39 mg/Dl	52 mg/dL
LDL^b^ cholesterol	0-99 mg/dL	115 mg/dL
Total cholesterol	100-199 mg/dL	185 mg/dL
Triglycerides	0-149 mg/dL	164 mg/dL

^a^HDL: high-density lipoprotein.

^b^LDL: low-density lipoprotein.

#### Semistructured Interview

To further understand patients’ information needs and challenges related to understanding laboratory test results, we conducted semistructured interviews with 13 people who had participated in the previous survey (Table 3). The interviews were conducted via Skype by 2 trained researchers and lasted from 30 min to 1 hour. During the interview, we asked participants to walk through the process of receiving a test result and probed them with questions about their understanding of the results, information needs, challenges faced, perceptions of patient-provider communication, and facilitators and barriers to using the patient portal. All the interviews were audio recorded with the participants’ permission and transcribed verbatim. All the participants received a US $20 Amazon gift card as compensation for their time.

**Table 3:** Characteristics of interview participants.

ID	Gender	Age (years)	Ethnicity	Occupation	Technology proficiency level	Health literacy level
Patient 1	Female	50-64	White	Unemployed	4	5
Patient 2	Male	50-64	African American	Retired	5	4
Patient 3	Female	50-64	White	Retired	5	5
Patient 4	Male	50-64	White	Technical analyst	5	5
Patient 5	Male	26-49	White	IT clerk	5	3
Patient 6	Male	26-49	White	Dog trainer	4	3
Patient 7	Female	50-64	White	Landscaper	3	4
Patient 8	Female	50-64	White	Executive assistant	5	5
Patient 9	Male	18-25	Asian	Medical assistant	4	4
Patient 10	Male	26-49	Asian	Visual artist	5	5
Patient 11	Female	26-49	White	Office assistant	2	4
Patient 12	Female	26-49	White	Sales	5	5
Patient 13	Female	26-49	White	Unemployed	4	4

### Data Analysis

We used descriptive statistics to analyze the survey data. Correlations between the variables were evaluated using the chi-square test or Fisher exact test where appropriate. In particular, we tested the association between several intrinsic factors (eg, age, gender, educational background, technology proficiency, health literacy, and normality of results) and participants’ perceptions of using patient portals to review laboratory results. All the tests were 2-sided, and the level of significance was set at 0.05. Statistical analysis was conducted using IBM SPSS 25.0. Detailed quantitative results are provided in Tables 4 and 5 and in the tables in Multimedia Appendix 3.

**Table 4:** Patient perceptions of interpreting test results and the association to normality of laboratory test results and health literacy (n=199).

Patient perception		Total^a^, n (%)	Normality of results (*P* value)	Health literacy (*P* value)
**Did your physician communicate with you about the test results before you viewed the results?**				
	Yes	112 (56.3)	.001^b^	.18
	No	87 (43.7)	.001^b^	.18
**Did you understand the result?**				
	Yes	120 (60.3)	.12	.03^c^
	No	9 (4.52)	.12	.03^c^
	Not sure	70 (35.2)	.12	.03^c^
**How did you know the test result was abnormal or normal?^d^**				
	Visual cue on patient portals	86 (43.2)	.98	.02^c^
	Clinician’s explanation	104 (52.3)	.60	.21
	Personal knowledge	70 (35.2)	.90	.50
	Other	11 (5.5)	.59	.18
**What kinds of confusion did you have?^d^ (n=136)**				
	Normal versus abnormal laboratory result	43 (31.6)	.84	.64
	Medical terminology	83 (61.0)	.37	.01^c^
	Meaning of the results	54 (39.7)	.66	.10
	Effects on my future health	47 (34.6)	.80	.61
	Treatment option	27 (19.9)	.69	.68
	Other	3 (2.2)	.87	.16
**How did you feel when you saw the result?**				
	Negative	29 (14.6)	<.001^e^	.001^b^
	Positive	144 (72.4)	<.001^e^	.001^b^
	Indifferent	26 (13.1)	<.001^e^	.001^b^
**What actions have you taken after viewing your test results?^d^**				
	Spoke with family and/or friends	101 (50.8)	.86	.45
	Looked up information on the web	123 (61.8)	.79	.60
	Posted questions in online health forums	22 (11.1)	<.001^e^	.89
	Created graph of results	9 (4.5)	.30	.41
	Emailed doctor	19 (9.6)	.19	.27
	Called doctor	22 (11.1)	.77	.88
	Made doctor’s appointment	34 (17.1)	.09	.09
	Other	11 (5.5)	.89	.27
**Do you need more information to interpret test results?**				
	Agree	118 (59.3)	.005^b^	.01^c^
	Neither agree nor disagree	58 (29.2)	.005^b^	.01^c^
	Disagree	23 (11.6)	.005^b^	.01^c^
**What types of information do you need?^d^** **(n=119)**				
	Prognosis	55 (46.2)	.007^b^	.68
	Treatment options	79 (66.4)	.86	.11
	What to do or ask	57 (47.9)	.50	.74
	Lifestyle changes	42 (35.3)	.50	.72
	Connecting with local support group	4 (3.4)	.57	.48
	Other	1 (0.8)	.45	.27

^a^In total, 4 participants could not remember the normality of their test results. These 4 participants were excluded from this analysis; therefore, the total number of cases was 199.

^b^Results with *P* value<.01.

^c^Results with *P* value<.05.

^d^Participants can select more than one option for those questions.

^e^Results with *P* value<.001.

**Table 5:** The association between 2 factors (health literacy and technology proficiency) and patient perceptions of using patient portals to understand and review laboratory test results (N=203).

Patient perceptions		Participants, n (%)		Health literacy (*P* value)		Technology proficiency (*P* value)	
**I am comfortable with using patient portals to review my** **laboratory** **results**							
	Agree		185 (91.1)		.06		.09
	Neither agree nor disagree		18 (8.9)		.06		.09
	Disagree		0 (0.0)		.06		.09
**I never had any trouble checking my test results on the patient portal**							
	Agree		161 (79.3)		.38		.002^a^
	Neither agree nor disagree		25 (12.3)		.38		.002^a^
	Disagree		17 (8.4)		.38		.002^a^
**I find the patient portal can make me review my tests quickly**							
	Agree		187 (91.6)		.53		<.001^b^
	Neither agree nor disagree		15 (7.4)		.53		<.001^b^
	Disagree		1 (1.0)		.53		<.001^b^
**I find the patient portal is useful to understand my** **laboratory** **results**							
	Agree		166 (81.3)		.15		.02^c^
	Neither agree nor disagree		30 (14.8)		.15		.02^c^
	Disagree		7 (3.9)		.15		.02^c^
**I have used the resources provided by patient portals to understand my results**							
	Agree		131 (64.0)		.03^c^		.22
	Neither agree nor disagree		40 (19.7)		.03^c^		.22
	Disagree		32 (16.3)		.03^c^		.22
**Is there anything that would make the portal better for you?^d^**							
	Make it more user-friendly		61 (29.1)		.70		.82
	Allow me to send a message to my physician		83 (40.0)		.04^c^		.009^a^
	Include a health encyclopedia that contains more information about the test		99 (47.3)		.48		.32
	Provide timely test result explanation and follow-up instructions		108 (52.7)		.007^a^		.07
	Other		2 (1.0)		.03^c^		.53

^a^Results with *P* value *P*<.01.

^b^Results with *P* value *P*<.001.

^c^Results with *P* value *P*<.05.

^d^Participants can select more than one option for those questions.

We used thematic analysis [36] to analyze the interview data. We first reviewed the interview transcripts to obtain an overview of the context. In the subsequent stage, we transferred data into NVivo (QSR International), a program for organizing, storing, and manipulating qualitative data. Two authors conducted open coding over the interview data and met regularly to discuss codes and code definitions, consolidate and refine codes until they reached consensus. In the second round of analysis, coded data were grouped under themes using affinity diagrams [37]. Themes describing the challenges and information needs of interpreting laboratory results and perceptions of receiving and reviewing results via current delivery mechanisms emerged after the second round of coding.

## Results

### Challenges in Comprehending Laboratory Test Results

The results showed that only 55.2% (112/203) of participants reported that they were contacted by their physician before reviewing the results in the patient portal, suggesting that it is not always the case for patients to receive timely notification and explanation of the results from their physician. One participant explained: “I didn’t actually talk to the doctor until probably like a week after that, because I wasn't able to get a hold of them” (P10). This finding is consistent with previous work stating that patients usually did not receive any explanatory information from their physician when they received the result [38]. Compared with patients who received normal results, those with abnormal results were more likely to be contacted (*P*=.001). When the physician explanations were absent, it is surprising to see that some participants chose not to bother their physician:

We have these mega practices, they’re incredibly busy, and I think if I did, I’d probably be afraid of being labeled as a trouble patient, and you don't want to be a trouble patient. I want to reserve those chips for when I really need them.

P3

We also found that participants with relatively high technology proficiency were more likely to possess positive opinions regarding the usefulness of patient portals (eg, the patient portal was useful in reviewing the test quickly [187/203, 91.1%; *P*<.001] and understanding their laboratory results [166/203, 81.3%; *P*=.02]). In a similar vein, participants with relatively high health literacy would be more likely to use resources provided by patient portals to make sense of the laboratory results (131/203, 64%; *P*=.03). These findings align with previous research showing that there is a digital divide in the use and adoption of health technologies—people with higher literacy and numeracy can benefit more from using health technologies to manage their health [19].

Finally, our participants indicated that existing patient portals lack sufficient and useful information for patients to understand their results: “In my particular case, I am trying to understand the meaning of the various test results. I didn't see anything on the patient portal that helped me with that at all” (P4). The lack of timely explanation from a primary care physician exacerbates this challenge, leaving patients entangled with how to interpret the meaning of their results accurately.

### Patients’ Confusion About Laboratory Test Results

Given these challenges, it is not surprising that many participants have difficulties understanding their laboratory results. We found that 70 survey participants (70/199, 35.2%) were unsure whether they understood their laboratory results. We believe this is because making sense of laboratory test results is a multilayered issue. Patients need to not only know whether they received a normal or abnormal result but also understand the meaning and indications of the test results. Despite participants reporting using different mechanisms to interpret the normality of test results (eg, visual cues on patient portals and personal knowledge), we found that 136 out of 203 participants (136/203, 67.0%) still reported having confusion about different aspects of their laboratory results, including medical terminology, reference range, the meaning of laboratory value, and the effects on their health care.

Participants deemed technical jargons, such as medical terminology and abbreviations, to be most confusing (83/136, 61.0%):

Just some of the specifics to the result. Like in the blood tests, words like neutrophils, or CBC, or bilirubin, however you pronounce that. Those very specific things that as a person who is not trained in medicine, I didn't understand without having to consult the internet.

P4

Although some patient portals have started implementing consumer-friendly vocabularies [39], this challenge persists—those explanations of medical terms may use additional medical jargon, which further confuses the patients. This perception was echoed by other participants who stated that patient portals overly use professional medical terms that are not easy to understand for lay individuals.

Our participants also had trouble differentiating between normal and abnormal laboratory results (43/136, 31.6%), in particular, whether a specific laboratory value falls into the normal range: “Some of the numbers don't even have a point of reference for me, so I don't understand that” (P12). In addition, some participants also reported confusion about some test results that are not in a numerical format, such as *false positive* and *nonreactive*. One participant explained:

I get what the HIV means. And then like you actually even click further to details and it didn't even say “Negative”, it is actually “Reactive”. That’s what I didn't understand. I didn't know what non-reactive meant.

P6

For those who received out-of-range test results, they reported that they struggled to understand how bad their abnormal results were and how alarmed they should feel.

Finally, participants expressed that they lacked an understanding of the meaning of the results (54/136, 39.7%) and the effects on their overall health care (47/136, 35.6%):

I don't know exactly what it (test result) meant clinically and for my overall health. I just kind of had a general idea. So I’m undergoing cancer treatment and so they’re checking my white blood cell counts. Those were low, but I don't really understand are they too low? I don't know necessarily what all of the things are. Like the neutrophils and I don't know what all of that means if it’s out of range.

P13

### Unmet Information Needs

A considerable number of participants (119/203, 58.6%) expressed an interest in obtaining more pertinent information in addition to that provided by patient portals to interpret their test results. In particular, both normality of the laboratory test (*P*=.005) and health literacy (*P*=.01) were significantly associated with this requestparticipants with abnormal results (43/58, 74.1%) and relatively high health literacy were more interested in receiving additional information (116/192, 60.4%). Some participants would like to know more generic information, such as reference range and diagnostic abilities of a specific test (eg, “I guess I liked to know an explanation of what the normal levels should be [...] and what each of the tests was testing” [P9]). In contrast, others preferred personalized and contextual information (eg, “If it was more personalized based on a patient’s history, that would definitely be more helpful because then it would be something you could really know [if you] need to act on this or not” [P12]). The types of most needed personalized information include treatment options, what to do or ask, prognosis, and lifestyle changes.

More specifically, many of the participants (79/119, 66.4%) would like to know more information about treatment options, including medication and medical procedures: “I want to know something like treatment options or medications” (P8). Of particular interest here is that 35.5% (50/119) of the participants whose results were normal also expressed an interest in knowing the treatment options. One possible explanation is that the participants were asked to report the normality of their most recent laboratory test result. Therefore, some participants may receive normal results for the latest test, but they may have received an abnormal result before. We speculate that these participants expressed their general preferences and information needs not only based on the most recent result but also on their overall experience.

Our participants also needed assistance in deciphering the meaning of the results and understanding the potential prognosis (56/119, 47.1%):

I would like to see a little more information as far as what might be causing it if there is a result that’s not normal. I would like to see a little more as far as what possibly could be there.

P12

Interestingly, laboratory test normality was strongly associated with whether participants would like to know prognosis information (*P*=.007).

There was also a demand to be informed about what they could do to cope with the bothersome symptoms they were experiencing or what they should ask the doctor during the next clinic visit (58/119, 48.7%): “I liked to know suggestions of what I should do. […] If I get an abnormal result, I would be wondering what I could be doing to improve” [P9]. In particular, people also wanted to know whether changing their lifestyle (eg, diet and exercise) could be of any help (42/119, 35.3%). Knowing such information could help them better manage their health care (eg, take actions to address elevated creatinine levels) and make informed decisions (eg, whether and when to see a doctor).

### Emotional Aspect of Viewing Laboratory Results

Despite only 29 out of 203 participants (29/203, 14.3%) reporting the experience of negative emotions (eg, concerns, anxiety, and frustration) while viewing the results, we noticed that laboratory result normality was strongly associated with feelings (*P*<.001). This is consistent with previous work [23]—participants with abnormal results were more likely to experience negative emotions (25/58, 43.1%). One participant shared her story and expressed the necessity of getting emotional support when the result is abnormal:

I was diagnosed having cancer. So I need to do a bunch of lab tests regularly to keep track of everything. I was kind of scared every single time when I was checking the test results online. I hoped to see improved numbers. But they were not always good and when that happened, my level of anxiety skyrocketed in a second. It can be very tough.

P10

### Actions Taken

To fulfill their knowledge gap and emotional needs, participants took several actions, including speaking with family and/or friends (101/203, 49.6%), looking up information online (124/203, 61.1%), posting questions in online health care forums (23/203, 11.3%), emailing and/or calling a doctor (42/203, 20.7%), and making a doctor’s appointment (35/203, 17.2%). These findings show that many people sought information about their results from sources other than their physician [23].

In particular, laboratory test normality was significantly associated with posting questions online (*P*<.001)—participants with abnormal results would be more likely to post questions in online health care forums compared with those receiving normal results (14/58, 24% vs 8/141, 5.7%). By doing so, they were able to prepare questions or sought relevant information before their next clinic visit. However, several participants reported finding misleading and/or disturbing information on the internet (eg, “once you hit the forums, you often get crazy time”). A few participants also mentioned that online searches helped clarify medical terms but did not yield much useful information for comprehending specific results (eg, “It’s too general. It’s not helpful”).

### Strategies to Improve Patient Portals

When asked how to make the patient portal better and useful for the comprehension of laboratory results, participants ranked “provide timely test result explanation and follow-up instructions” (108/203, 52.7%), “include a health encyclopedia that contains more information about the test” (99/203, 47.3%), and “allow me to send a message to my physician” (83/203, 40.8%) as the top 3 needed features.

Our participants also emphasized the importance of making the patient portals “more user-friendly” (61/203, 29.1%). For instance, patient portals should be designed with patients in mind rather than for clinician interpretation only, as one participant stated:

So maybe making it less like I’m looking at the system that the doctor would be using and more something that’s designed with the patient in mind and understanding who the patient is and not somebody who’s going to use like the very specific language.

P6

They further suggested that visualizing historical results (“What I really, really think needs to be done is the ability to track your abnormal results over time”) and using lay terms to explain the result (“make it like a 10-year-old would understand”) could be very useful in improving the user experience of patient portals.

The design of patient portals should also take into consideration marginalized user groups, including patients with disability and older adult patients, to minimize the disparities in the use of patient portals [40]. One participant explained why this issue is important to address:

I noticed with patient portals is they’re not very accessible to people with vision issues. It doesn't have to be fancy, but a little button that says “please push F to read this for them”, and then they could hear it. [...] or maybe the doctor gives a specialized URL and he like “hey, go to this URL, and it will be for blind assist” [...] I would say it needs to be a little bit more use-friendly as far as older folks go. [...] So since the world is having a national aging problem, it should be geared more for grandma to understand it.

P5

Finally, it is interesting to see that several participants described the potential of incorporating artificial intelligence (AI) technology into patient portals so that their data can be used to generate more personalized medical information:

I know right now a big part of medicine is going to be AI where you actually interact with artificial intelligent agents. They’ll probably have your information right there and it’ll probably make it a lot easier for you to get a lot of your answers because they’ll be accessing your information right there and giving you the answers to your questions.

P2

## Discussion

### Principal Findings

Although health care providers are expanding patients’ access to laboratory test results through patient portals, less than one-third of Americans had accessed this information online [7,23]. We conducted this study to better understand lay people’s perceptions about reviewing and comprehending laboratory test results through patient portals, including their perceived challenges and confusions as well as informational, emotional, and technological needs. The challenges faced by patients when reviewing laboratory test results (eg, lack of explanatory information from physicians and lack of useful information on patient portals) inevitably caused confusion about different aspects of their laboratory report, such as the meaning of laboratory values and the indications on their health care. To cope with these challenges and confusion, our participants expressed the urgent need to obtain a variety of information to comprehend laboratory results, including treatment options, what to do or ask, prognosis, and lifestyle changes. The desired enhancements of patient portals include providing timely explanations and educational resources (eg, a health encyclopedia), increasing usability and accessibility, and incorporating AI-based technology to provide personalized recommendations. Finally, we found that several intrinsic factors (eg, laboratory result normality, health literacy, and technology proficiency) had significant impacts on people’s perceptions of using portals to view and interpret laboratory results. For example, participants with relatively high health literacy were more likely to understand the meaning of test results and use resources provided by patient portals to make sense of their data, while people with relatively high technology proficiency tended to agree that the patient portal was easy to use and very helpful in reviewing and understanding their laboratory values. These findings highlight significant disparities in laboratory result comprehension and patient portal use among certain groups of the population.

### Design Implications

At present, the design of test results in patient portals seems to assume that patients have sufficient medical knowledge about their test results. In addition, patients often did not receive explanatory information at the time they received the result. One primary reason is that many physicians would prefer explaining results during a face-to-face clinical encounter to avoid any potential miscommunications [41]. However, on the other hand, patients are increasingly interested in accessing and interpreting their results. Many of them would search online to conduct their research [23,25,26,28]. Our participants articulated that internet searches were helpful in clarifying medical terms; however, the online information was not always reliable, and they even found contradicting or disturbing information on the internet, which caused further confusion and anxiety [28]. It is therefore crucial to provide more useful, credible, and actionable information to aid patient understanding, something current patient portals do not provide [23].

With regard to the types of information to provide, our results indicate that people have different information needs, including generic information (eg, reference range and diagnostic abilities of a specific test) and personalized or contextual information (eg, treatment options, prognosis, and what to do or ask next). This variety of information needs may be due to different levels of health literacy and numeracy that people have and whether patients receive normal or abnormal results [19]. For example, some patients may not be literate enough to understand the medical terminologies or normal ranges of a test, whereas others need help with more complicated issues, such as interpreting the laboratory results in the context of their medical history. It is, therefore, important to assess the patient’s knowledge level and provide corresponding support based on patients’ characteristics [42,43]. One way to accomplish this is to allow patients to add their own notes about their background (eg, health literacy, medical condition, etc) to a specific section in their patient portals to enable automatic assessment and customized support. It may also be useful to provide links to consumer-friendly and trusted information sources (eg, entries in MedlinePlus) to assist those who do not have high levels of health literacy and numeracy to read and interpret laboratory content [42]. We also noticed that people with abnormal results were more likely to ask for prognosis information. This indicates that providing interpretation and other advanced information (eg, prognosis, treatment options) to patients who received abnormal results at the time of portal release should be considered a best practice [15], as it can support patients’ information needs and reduce any anxiety they may experience.

We are not arguing that we should provide all kinds of information and support to patients at one time because they may overwhelm patients and create even more confusion. Instead, we believe that the delivery of additional information should be tailored to patients’ individual preferences, that is, patients should have options to decide whether they want to receive additional information in patient portals and what the information is at the time of ordering the test. Those who do not want to receive additional information can opt out and wait until their physician to provide explanations. This practice aligns with the SPIKES guideline (S: setting, P: perception, I: invitation, K: knowledge, E: empathy, S: summarize) of delivering health information to patients [44]. We also see design examples where patients can click buttons or hyperlinked texts to see additional information [29]. Presenting additional information in *pop-ups* is expected to not only ease the navigation of the interface but also empower patients to decide whether they would like to see further details.

A lot of the challenges in result comprehension faced by patients can, in part, be attributed to the interface design of patient portals. We examined how patient portals can be improved to support understanding. In line with previous work [29], we conclude that merely providing access to abundant data is insufficient; instead, the patient portal should employ user-centered design strategies to help patients better interpret and manage their test results. For example, the test results should be displayed to patients in ways they can comprehend and grasp the meaning of each test result to inform their decision making and subsequent actions. For example, it would be much easier for patients to interpret their results by delivering intuitive *gist* messages (eg, “your result was normal,” “your cholesterol levels were optimal”) when presenting numerical values [15,35]. Furthermore, as our participants described, they struggled to map a particular abnormal test result to its clinical meaning. Although most patient portals provide patients with a standard reference range to discriminate between normal and abnormal test results, patients cannot necessarily understand how bad the result is and if any urgent action is needed. The literature has suggested that it might be useful to provide an additional reference point to indicate how far outside the standard range that values become clinically concerning [21]. By doing so, it will help patients better distinguish different types of abnormal test results, that is, slightly out-of-range results versus extreme values, and in turn reduce the perceived urgency of abnormal results that do not require immediate clinical action [21].

Furthermore, our participants discussed the possibility of incorporating AI technology into patient portals to make use of their clinical data to generate more personalized medical information. Indeed, with the recent rise in the capabilities of AI, it is expected that AI-empowered health care systems can intelligently search, retrieve, and present pertinent information to patients within the context of their medical history [45]. For example, although many hospitals and physicians are still using the 2.5 or 3.0 mU/L cutoff (2.5 mU/L in the first trimester and 3.0 mU/L in the second and third trimesters) for the thyroid-stimulating hormone during pregnancy, a recent study pointed out that these cutoffs are too low and may lead to overdiagnosis or even overtreatment [46]. The AI-driven patient portals should be able to recommend such relevant and up-to-date medical evidence to patients based on their test results. Patient portals may embody AI technology to change the landscape of how people receive laboratory results and seek medical advice.

Finally, although our participants wanted to be informed about the meaning of diagnostic results, needless anxiety and negative emotions may arise when communicating *abnormal* diagnostic results without the presence of a medical professional. In particular, we found that patients with abnormal results were more likely to experience negative emotions. Therefore, it is necessary to provide additional support or resources for patients to mitigate emotional stress when communicating sensitive results through patient portals. For example, patients should have options to determine whether they want to receive concerning results via the portal. This could ensure that patient portals deliver potentially stressful health information in a more empathic manner [47]. In addition, previous work has shown that patient navigators can reduce negative emotions, such as, anxiety, for patients who receive abnormal mammography while waiting for follow-up testing [48].

However, implementing these design suggestions is challenging. This is because current patient portals lack consistency in the design for communicating laboratory test results across different portal vendors, which not only frustrates patients but also creates barriers for incorporating *best practices* and new features to different portals [23,49]. Therefore, an urgent research agenda is determining how to develop standards and guidelines and encourage portal providers to incorporate them to enhance patients’ understanding of their test results.

In brief, when communicating test results via portals, it is necessary to include information about the test itself (eg, test capability, the purpose of test), easy-to-understand explanations of medical jargon, the results in the context of the patient’s health, directions for next steps, and specific educational resources [35,50,51]. Furthermore, designers and developers of patient portals should embody user-centered approaches to significantly improve the design of patient portals to present information in a more meaningful way [29,52]. Finally, national test result notification and interface design standards for patient portals should be developed to ensure interoperability and consistency in features across portal vendors. Health policies should be enacted to support these strategies [23].

### Limitations and Future Research

A few limitations should be noted. First, our survey and interview participants self-reported their experiences with using patient portals to view laboratory results. Some of our respondents may have difficulties recalling such experiences. As described in the Methods section, we provided a commonly seen laboratory test to participants to cope with this issue. Second, our results may not generalize to all types of patients because we recruited our participants online and our sample inevitably consisted of a large majority of participants who self-reported to have medium to high levels of technology proficiency and health literacy. This limitation of participant recruitment could affect the generalizability of the results because there is a lack of a great representative of marginalized populations who can benefit most from this research. In our future work, we will include more marginalized populations (eg, less literate people, older adults) and examine how to improve their understanding of laboratory results. Third, this study did not investigate which patient portal our participants used. However, we believe it has a limited impact on our results as many patient portals share similar characteristics, such as displaying results in a tabular format with a reference range. Finally, we will design and prototype a new interface of patient portals for test results based on our findings. We will involve different types of patients with various characteristics and use a multiphased, user-centered approach combined with rapid prototyping and formative evaluation.

### Conclusions

In conclusion, our findings suggest that there are challenges for patients to comprehend their laboratory test results through the patient portal. This is mainly because of the lack of communication with physicians and the lack of support and useful information in current patient portals. Several factors, including participants’ health literacy and technology proficiency as well as laboratory result normality, have impacts on people’s perceptions of using patient portals to understand the laboratory results. Our participants have a variety of information needs, ranging from general information (eg, reference range and diagnostic abilities of a specific test) to personalized information (eg, treatment options, prognosis, and what to do or ask next). Participants also emphasized the importance of improving patient portals to better meet their needs. This study contributes to an empirical, in-depth understanding of the challenges and needs of patients in comprehending laboratory test results and informs strategies and design implications for informatics tools to promote patients’ comprehension of test results.

## Appendix

Multimedia Appendix 1 Survey questionnaire.

Multimedia Appendix 2 Interview protocol.

Multimedia Appendix 3 Patient perceptions of interpreting test results and the association to health literacy and normality of lab test and patient perceptions of using patient portal and the association to health literacy and technology proficiency.

## Abbreviations

AI: artificial intelligence
CMA: chromosomal microarray
MTurk: Amazon Mechanical Turk
NIH: National Institutes of Health
RQ: research question
