# Supplementary material for: Patient Challenges and Needs in Comprehending Laboratory Test Results: Mixed Methods Study
Source: J Med Internet Res. 2020 Dec 7;22(12):e18725. doi: 10.2196/18725 (PMC7752528; doi:10.2196/18725)
Supplement: Multimedia Appendix 1 [file jmir_v22i12e18725_app1.pdf]

Survey _ Understanding Clinical Lab Test Results

Notes: Questions with a hash tag (#) are displayed only when certain response is provided. Questions with an asterisk (*) allow participants to select more than one options.

Screen Questions

Did you take any clinical lab test (e.g., blood test, urine test, MRI, CT-scan) over the past six months?

- Yes
- No

Have you ever used patient portals implemented within electronic medical record to view your test results?

- Yes
- No

Notes: If the answer is "No" to either of the screen questions, the Mechanical Turk (MT) worker is not eligible for this survey. The following message will be displayed and the survey will end.

"Thank you for your interest in taking this survey! However, we are looking for people who have the experience of taking clinical lab tests and using patient portals to view the results. Thus, we regret to tell you that you are NOT eligible for taking this survey. Thanks again for your interest!"

If the answer is "Yes" to both screen questions, the MT worker will be directed to review and agree to the consent form. And then the worker will start taking the rest of the survey.

Demographic Information:

What is your gender

- Female
- Male
- Other

How old are you?

- 18-25 years
- 26-49 years
- 50-64 years
- 65 and older

What is your race/ethnicity?

- Asian or Pacific Islander
- Black/African American
- Hispanic/Latino

- American Indian/Native American
- White/Caucasian
- Other (please specify)

What is your highest degree or level of school you have completed? If currently enrolled, highest degree received.

- Less than a high school diploma
- High school degree or equivalent
- Associate degree
- Bachelor's degree
- Master's degree
- Doctorate degree
- Other (please specify)

What is your employment status?

- Unemployed
- Part time
- Full time
- Other (please specify)

(*follow-up questions, only shows when participant answered "Part time" or "Full time*")
#If you are employed, what is your occupation?

#What best describes your industry?

- Government
- Health Care
- Education
- Finance
- Information Technology
- Other (please specify)

How do you rate your health literacy? Health literacy means "The degree to which an individual has the capacity to obtain, communicate, process, and understand basic health information and services to make appropriate health decisions."

1- low; 2-low to medium; 3-medium; 4-medium to high; 5-high

How do you rate your technology literacy? Technology literacy means "The ability of an individual, working independently or with others, to use tools, resources, processes, and systems responsibly to access and evaluate information, and use that information to make informed decisions."

1- low; 2-low to medium; 3-medium; 4-medium to high; 5-high

Patient's Perceptions

Please reflect on the test results you received most recently. If you have no direct access to your results, you can use the sample data (lipid panel) provided here to answer the following questions

Lipid profile:

	**Reference Range**	**Your Results**
HDL Cholesterol	>39 mg/dL	52 mg/dL
LDL Cholesterol	0-99 mg/dL	115 mg/dL
Total Cholesterol	100-199 mg/dL	185 mg/dL
Triglycerides	0-149 mg/dL	164 mg/dL

How many times did you view your test results over the past six months?

- 1 time
- 2-5 times
- 6-10 times
- >10 times

When were test results last viewed?

- Within last month
- 2-3 months ago
- 4-6 months ago
- Don't remember

Did you get abnormal test results?

- Yes
- No

Did your physician communicate with you about the test results before you viewed the results? (For example, your doctor may write a note to you or call you explaining the result)

- Yes
- No

Did you understand the result?

- Yes
- No
- Somewhat understand

(*follow-up question, only shows when participant answered "No" or "Somewhat understand"*)
#*What (if any) kinds of confusion did you have?

- Normal vs. abnormal lab result (reference range)
- Medical terminology
- Meaning of the results

- Effects on my future health
- Treatment option
- Other (please specify)

*How did you know the test result was abnormal or normal?

- Visual cue on patient portals (range, flag, color, bold)
- Clinician's explanation/note
- Personal knowledge/experience
- Other (please specify)

How did you feel when you saw the result?

- Negative (concerned, confused, anxious, scared, frustrated)
- Positive (happy, curious, relieved, relaxed, satisfied)
- Indifferent

*What (if any) actions have you taken after viewing your test results?

- Spoke with family and/or friends
- Looked up information online
- Posted questions in online healthcare forums
- Created graph of results
- Emailed doctor
- Telephoned doctor
- Made doctor's appointment
- Other (please specify)

I need more information than those provided in patient portals to fully understand my test results.

- Agree
- Neither agree nor disagree
- Disagree

(*follow-up question, only shows when the above question is answered "Agree"*)
#*What types of personalized information or advice do you need?

- Prognosis based on my medical history
- Treatment options
- Questions to ask when meeting my doctor
- Lifestyle changes
- Connecting with local support group
- Other (please specify)

Use of Patient Portals

I am comfortable with using patient portals to review my lab results.

- Agree
- Neither agree nor disagree
- Disagree

I never had any trouble checking my test results on the patient portal?

- Agree
- Neither agree nor disagree
- Disagree

(*Follow-up question, only shows when the above question is answered "disagree"*)
What kinds of trouble did you encounter when using patient portal to check your test results?

I find the patient portal can make me review my tests quickly.

- Agree
- Neither agree nor disagree
- Disagree

I find the patient portal is useful to understand my lab results.

- Agree
- Neither agree nor disagree
- Disagree

I find the patient portal provides enough useful resources to understand my lab results.

- Agree
- Neither agree nor disagree
- Disagree

I have used the resources provided by patient portals to understand my lab results, such as explanations of medical terminology.

- Agree
- Neither agree nor disagree
- Disagree

*Is there anything that would make the portal better for you?

- Make it more user-friendly
- Allow me to send a message to my physician
- Include a health encyclopedia that contains more information about the test
- Provide timely test result explanation and follow-up instructions
- Other (please specify)

Thanks again for taking the survey!

We would like to use this opportunity to invite you to participate in our follow-up interview study. Your participation is greatly appreciated and your time will be compensated.

Please contact us (zzhang@pace.edu) if you are interested in participating in our follow-up interview study.
