# Supplementary material for: Patient Challenges and Needs in Comprehending Laboratory Test Results: Mixed Methods Study
Source: J Med Internet Res. 2020 Dec 7;22(12):e18725. doi: 10.2196/18725 (PMC7752528; doi:10.2196/18725)
Supplement: Multimedia Appendix 2 [file jmir_v22i12e18725_app2.pdf]

Interview Guide

Equipment/Material

- Consent form
- Audio-recorder
- Paper, Pens/Markers
- Prototype
- Conferencing Tool

Prior to interview: Consent form is sent to the participant to review in advance.

Introduction: Introduce self, welcome, and thank participants for agreeing to participate in our study.

Explain the purpose The current technologies for managing and reporting clinical data, such as patient portals, may have been designed to present clinical data to patients in the same way as it is shown to doctors, or do not provide enough, or the right, support to aid patient understanding their personal healthcare data, such as lab test results. This research aims to develop applications to explain personal health information in meaningful ways and provide personalized advice (e.g., treatment options) based on patients' medical context. In this interview, we want to understand your perceptions of current patient portals' presentation of clinical data and challenges in understanding such data.

Consent: Explain informed consent.

Remind the participant that the interview will be audio recorded, that participation is voluntary, that they have the right to withdraw at any point. During the interview, you are free to decline to answer any questions you find uncomfortable or you don't wish to.

Compensation: After the participation, $20 amazon e-gift card will be sent to the participant via email.

Ask if they have any questions about the study.

Sign if not yet done and collect consent form.

******Audio recording will start here.**

Questions:

<u>Information needs for understanding clinical data</u>

1. Could you share your most recent experience using patient portals to view your clinical information, such as lab test results, radiology reports, etc.?

   — How often

   — Last time

   — For what reason

2. Did you understand the meaning of the results?
   a. If the answer is "yes", then ask

   — Did you get normal or abnormal test results?

   — How did you know the meaning of the test results?

   b. If the answer is "no", then ask

   — What kinds of confusion did you have?

3. Did you have trouble understanding the terminologies and medical language in your lab test results?

4. What do you want to know after viewing your results (such as meaning of test results, treatment options, prognosis)?

5. What (if any) actions have you taken after viewing your test results?

6. Did you get timely and meaningful explanations regarding the results from your doctor?
   a. If the answer is "yes", then ask

   — When did you get the explanations from your doctor (before vs. after viewing the results)? And through what channel (email, phone call, etc.)?

   — Did you find your doctor's explanations sufficient for you to understand your health status? Any concerns?

   b. If the answer is "no", then ask

— Were you expecting explanations from your doctor?

— Are you comfortable with receiving test results without doctor explanation?

7. What, if any, challenges or concerns do you have in communicating with your doctor?

Patient Portal

8. Do you have any concerns with using patient portals to review your clinical data?
    a. If the answer is “yes”, then ask

    — What kinds of concerns or problems do you have with using patient portals?

    b. If the answer is “no”, then ask

    — Are you comfortable with using patient portals?

    — Have you ever had any trouble checking your lab results on the patient portal?

9. Do you find the patient portal is useful to understand your lab results? Do you feel the patient portal provides enough useful resources and information?

10. Have you ever used the information or resources provided by patient portals (e.g., explanations of terminology) to understand your results?

11. Have you ever used patient portal to communicate with your doctor? Do you find it is useful?

12. Do you find the patient portal is easy to use?

13. Is there anything that would make the portal better for you? What new features would you like to see?

Wrap-Up Discussion

Was there anything about the project that was confusing?

Is there anything that we should have asked you or is there anything else you would like to discuss?

*****Audio recording will end here.**

End of interview
