# Supplementary material for: Patient Challenges and Needs in Comprehending Laboratory Test Results: Mixed Methods Study
Source: J Med Internet Res. 2020 Dec 7;22(12):e18725. doi: 10.2196/18725 (PMC7752528; doi:10.2196/18725)
Supplement: Multimedia Appendix 3 [file jmir_v22i12e18725_app3.docx]

**Multimedia Appendix Table 1: Patient perceptions of interpreting test results and the association to normality of lab test results.**

| Patient perceptions | Total (n = 199), n(%) | Normality of Results | | *P* value |
| --- | --- | --- | --- | --- |
|  |  | Normal (n=141) | Abnormal (n=58) |  |
| **Did your physician communicate with you about the test results before you viewed the results?** |  |  |  | 0.00112** |
| Yes | 112(56.28) | 69(48.9) | 43(74.1) |  |
| No | 87(43.72) | 72(51.1) | 15(25.9) |  |
| **Did you understand the result?** |  |  |  | 0.121 |
| Yes | 120(60.3) | 79(56.03) | 41(70.69) |  |
| No | 9(4.52) | 8(5.67) | 1(1.72) |  |
| Not sure | 70(35.18) | 54(38.3) | 16(27.59) |  |
| **How did you know the test result was abnormal or normal?**† |  |  |  |  |
| Visual cue on patient portals | 86(43.22) | 61(43.26) | 25(43.1) | 0.984 |
| Clinician’s explanation | 104(52.26) | 72(51.06) | 32(55.17) | 0.598 |
| Personal knowledge | 70(35.18) | 50(35.46) | 20(34.48) | 0.895 |
| Other | 11(5.53) | 7(4.96) | 4(6.9) | 0.588 |
| **What kinds of confusion did you have?**†  **(n = 136)** |  |  |  |  |
| Normal vs. abnormal lab result | 43(31.62) | 31(21.99) | 12(20.69) | 0.84 |
| Medical terminology | 83(61.03) | 56(39.72) | 27(46.55) | 0.374 |
| Meaning of the results | 54(39.71) | 37(26.24) | 17(29.31) | 0.658 |
| Effects on my future health | 47(34.56) | 34(24.11) | 13(22.41) | 0.797 |
| Treatment option | 27(19.85) | 20(14.18) | 7(12.07) | 0.692 |
| Other | 3(2.21) | 2(1.42) | 1(1.72) | 0.872 |
| **How did you feel when you saw the result?** |  |  |  | <0.001*** |
| Negative | 29(14.57) | 4(2.84) | 25(43.1) |  |
| Positive | 144(72.36) | 118(83.69) | 26(44.83) |  |
| Indifferent | 26(13.07) | 19(13.48) | 7(12.07) |  |
| **What actions have you taken after viewing your test results?**† |  |  |  |  |
| Spoke with family and/or friends | 101(50.75) | 71(50.35) | 30(51.72) | 0.86 |
| Looked up information online | 123(61.81) | 88(62.41) | 35(60.34) | 0.785 |
| Posted questions in online health forums | 22(11.06) | 8(5.67) | 14(24.14) | <0.001*** |
| Created graph of results | 9(4.52) | 5(3.55) | 4(6.9) | 0.301 |
| Emailed doctor | 19(9.55) | 11(7.8) | 8(13.79) | 0.191 |
| Called doctor | 22(11.06) | 15(10.64) | 7(12.07) | 0.77 |
| Made doctor’s appointment | 34(17.09) | 20(14.18) | 14(24.14) | 0.09 |
| Other | 11(5.53) | 8(5.67) | 3(5.17) | 0.888 |
| **I need more information to interpret test results.** |  |  |  | 0.005* |
| Agree | 118(59.3) | 75(53.19) | 43(74.14) |  |
| Neither agree nor disagree | 58(29.15) | 44(31.21) | 14(24.14) |  |
| Disagree | 23(11.56) | 22(15.6) | 1(1.72) |  |
| **What types of information do you need?**† **(n=119)** |  |  |  |  |
| Prognosis | 55(46.22) | 42(29.79) | 13(22.41) | 0.0069** |
| Treatment options | 79(66.39) | 50(35.46) | 28(48.28) | 0.864 |
| What to do or ask | 57(47.9) | 38(26.95) | 19(32.76) | 0.497 |
| Lifestyle changes | 42(35.29) | 25(17.73) | 17(29.31) | 0.498 |
| Connecting with local support group | 4(3.36) | 2(1.42) | 2(3.45) | 0.566 |
| Other | 1(0.84) | 1(0.71) | 0(0) | 0.45 |

Note: *P<0.05 **P<0.01 ***P<0.001

† Participants can select more than one option for those questions.

ˆ Four participants couldn’t remember the normality of their test results. These four participants were excluded from this analysis, so the total number of cases is 199.

**Multimedia Appendix Table 2: Patient perceptions of interpreting test results and the association to health literacy.**

| Patient perceptions | Total (n = 203), n(%) | Health Literacy | | | | | *P* value |
| --- | --- | --- | --- | --- | --- | --- | --- |
|  |  | 1: Low  (n=1) | 2: Low-Medium  (n=10) | 3: Medium  (n=81) | 4: Medium-High  (n=67) | 5: High  (n=44) |  |
| **Did you understand the result?** |  |  |  |  |  |  | 0.029* |
| Yes | 121 (59.6) | 1(100) | 2(20) | 40(49.38) | 45(67.16) | 33(75) |  |
| No | 10 (4.9) | 0(0) | 1(10) | 6(7.41) | 2(2.99) | 1(2.27) |  |
| Not sure | 72 (35.5) | 0(0) | 7(70) | 35(43.21) | 20(29.85) | 10(22.73) |  |
| **How did you know the test result was abnormal or normal?**† |  |  |  |  |  |  |  |
| Visual cue on patient portals | 88 (43.3) | 1(100) | 8(80) | 28(34.57) | 27(40.3) | 24(54.55) | 0.019* |
| Clinician’s explanation | 105 (51.7) | 0(0) | 3(30) | 47(58.02) | 36(53.73) | 19(43.18) | 0.206 |
| Personal knowledge | 71 (35.0) | 0(0) | 4(40) | 23(28.4) | 26(38.81) | 18(40.91) | 0.495 |
| Other | 11 (5.4) | 0(0) | 1(10) | 2(2.47) | 3(4.48) | 5(11.36) | 0.179 |
| **What kinds of confusion did you have?**† **(n = 136)** |  |  |  |  |  |  |  |
| Normal vs. abnormal lab result | 43(31.62) | 0(0) | 3(30) | 14(17.28) | 17(25.37) | 9(20.45) | 0.643 |
| Medical terminology | 83(61.03) | 0(0) | 5(50) | 46(56.79) | 22(32.84) | 13(29.55) | 0.01* |
| Meaning of the results | 54(39.71) | 0(0) | 4(40) | 25(30.86) | 22(32.84) | 6(13.64) | 0.102 |
| Effects on my future health | 47(34.56) | 0(0) | 3(30) | 22(27.16) | 17(25.37) | 7(15.91) | 0.608 |
| Treatment option | 27(19.85) | 0(0) | 2(20) | 12(14.81) | 11(16.42) | 4(9.09) | 0.681 |
| Other | 3(2.21) | 0(0) | 1(10) | 0(0) | 1(1.49) | 2(4.55) | 0.16 |
| **How did you feel when you saw the result?** |  |  |  |  |  |  | 0.001*** |
| Negative | 29 (14.3) | 0(0) | 2(20) | 5(6.17) | 19(28.36) | 3(6.82) |  |
| Positive | 148 (72.9) | 0(0) | 6(60) | 64(79.01) | 44(65.67) | 34(77.27) |  |
| Indifferent | 26 (12.8) | 1(100) | 2(20) | 12(14.81) | 4(5.97) | 7(15.91) |  |
| **What actions have you taken after viewing your test results?**† |  |  |  |  |  |  |  |
| Spoke with family and/or friends | 101(0.5) | 1(100) | 4(40) | 36(44.44) | 38(56.72) | 22(50) | 0.452 |
| Looked up information online | 124(0.61) | 0(0) | 6(60) | 46(56.79) | 43(64.18) | 29(65.91) | 0.603 |
| Posted questions in online health forums | 23(0.11) | 0(0) | 1(10) | 10(12.35) | 6(8.96) | 6(13.64) | 0.887 |
| Made graph of results | 9(0.04) | 0(0) | 0(0) | 2(2.47) | 3(4.48) | 4(9.09) | 0.411 |
| Emailed doctor | 20(0.1) | 0(0) | 1(10) | 4(4.94) | 10(14.93) | 5(11.36) | 0.266 |
| Called doctor | 22(0.11) | 0(0) | 0(0) | 10(12.35) | 7(10.45) | 5(11.36) | 0.882 |
| Made doctor’s appointment | 35(0.17) | 0(0) | 1(10) | 8(9.88) | 14(20.9) | 12(27.27) | 0.091 |
| Other | 11(0.05) | 0(0) | 2(20) | 3(3.7) | 4(5.97) | 2(4.55) | 0.268 |
| **I need more information to interpret test results.** |  |  |  |  |  |  | 0.014* |
| Agree | 119 (58.6) | 0(0) | 3(30) | 58(71.6) | 32(47.76) | 26(59.09) |  |
| Neither agree nor disagree | 61 (30.0) | 0(0) | 5(50) | 17(20.99) | 25(37.31) | 14(31.82) |  |
| Disagree | 23 (11.4) | 1(100) | 2(20) | 6(7.41) | 10(14.93) | 4(9.09) |  |
| **What types of information do you need?**† **(n=119)** |  |  |  |  |  |  |  |
| Prognosis | 56 (47.1) | 0(0) | 1(10) | 24(29.63) | 17(25.37) | 14(31.82) | 0.683 |
| Treatment options | 79 (66.4) | 0(0) | 2(20) | 40(49.38) | 22(32.84) | 15(34.09) | 0.106 |
| What to do or ask | 58 (48.7) | 0(0) | 1(10) | 25(30.86) | 20(29.85) | 12(27.27) | 0.743 |
| Lifestyle changes | 42 (35.3) | 0(0) | 2(20) | 16(19.75) | 12(17.91) | 12(27.27) | 0.72 |
| Connecting with local support group | 4 (2.0) | 0(0) | 0(0) | 1(1.23) | 3(4.48) | 0(0) | 0.484 |
| Other | 1 (0.4) | 0(0) | 0(0) | 0(0) | 0(0) | 1(2.27) | 0.271 |

Note: *P<0.05 **P<0.01 ***P<0.001

† Participants can select more than one option for those questions.

**Multimedia Appendix Table 3: The association between health literacy and patient perceptions of using patient portals to understand and review lab test results**.

| Patient perceptions | Total (n = 203), n(%) | Health Literacy | | | | | *P* value |
| --- | --- | --- | --- | --- | --- | --- | --- |
|  |  | 1: Low  (n=1) | 2: Low-Medium  (n=10) | 3: Medium  (n=81) | 4: Medium-High  (n=67) | 5: High  (n=44) |  |
| **I am comfortable with using patient portals to review my lab results.** |  |  |  |  |  |  | 0.059 |
| Agree | 185 (91.1) | 1(100) | 7(70) | 71(87.65) | 64(95.52) | 42(95.45) |  |
| Neither agree nor disagree | 18 (8.9) | 0(0) | 3(30) | 10(12.35) | 3(4.48) | 2(4.55) |  |
| Disagree | 0 (0.0) | 0(0) | 0(0) | 0(0) | 0(0) | 0(0) |  |
| **I never had any trouble checking my test results on the patient portal?** |  |  |  |  |  |  | 0.383 |
| Agree | 161 (79.3) | 1(100) | 5(50) | 63(77.78) | 54(80.6) | 38(86.36) |  |
| Neither agree nor disagree | 25 (12.3) | 0(0) | 3(30) | 11(13.58) | 8(11.94) | 3(6.82) |  |
| Disagree | 17 (8.4) | 0(0) | 2(20) | 7(8.64) | 5(7.46) | 3(6.82) |  |
| **I find the patient portal can make me review my tests quickly.** |  |  |  |  |  |  | 0.529 |
| Agree | 187 (91.6) | 1(100) | 8(80) | 74(91.36) | 62(92.54) | 42(95.45) |  |
| Neither agree nor disagree | 15 (7.4) | 0(0) | 2(20) | 6(7.41) | 5(7.46) | 2(4.55) |  |
| Disagree | 1 (1.0) | 0(0) | 0(0) | 1(1.23) | 0(0) | 0(0) |  |
| **I find the patient portal is useful to understand my lab results.** |  |  |  |  |  |  | 0.15 |
| Agree | 166 (81.3) | 1(100) | 6(60) | 64(79.01) | 54(80.6) | 41(93.18) |  |
| Neither agree nor disagree | 30 (14.8) | 0(0) | 3(30) | 15(18.52) | 10(14.93) | 2(4.55) |  |
| Disagree | 7 (3.9) | 0(0) | 1(10) | 2(2.47) | 3(4.48) | 1(2.27) |  |
| **I find the patient portal provides enough useful resources to understand my lab results.** |  |  |  |  |  |  | 0.309 |
| Agree | 143 (70.4) | 1(100) | 5(50) | 58(71.6) | 43(64.18) | 36(81.82) |  |
| Neither agree nor disagree | 35 (17.2) | 0(0) | 4(40) | 14(17.28) | 12(17.91) | 5(11.36) |  |
| Disagree | 25 (12.4) | 0(0) | 1(10) | 9(11.11) | 12(17.91) | 3(6.82) |  |
| **I have used the resources provided by patient portals to understand my results.** |  |  |  |  |  |  | 0.03* |
| Agree | 131 (64.0) | 0(0) | 5(50) | 51(62.96) | 40(59.7) | 35(79.55) |  |
| Neither agree nor disagree | 40 (19.7) | 1(100) | 4(40) | 20(24.69) | 12(17.91) | 3(6.82) |  |
| Disagree | 32 (16.3) | 0(0) | 1(10) | 10(12.35) | 15(22.39) | 6(13.64) |  |
| **Is there anything that would make the portal better for you?**† |  |  |  |  |  |  |  |
| Make it more user-friendly | 61 (29.1) | 0(0) | 3(30) | 27(33.33) | 16(23.88) | 15(34.09) | 0.698 |
| Allow me to send a message to my physician | 83 (40.0) | 1(100) | 4(40) | 38(46.91) | 30(44.78) | 10(22.73) | 0.039* |
| Include a health encyclopedia that contains more information about the test | 99 (47.3) | 0(0) | 5(50) | 43(53.09) | 34(50.75) | 17(38.64) | 0.475 |
| Provide timely test result explanation and follow-up instructions | 108 (52.7) | 0(0) | 6(60) | 32(39.51) | 45(67.16) | 25(56.82) | 0.007** |
| Other | 2 (1.0) | 0(0) | 1(10) | 0(0) | 0(0) | 1(2.27) | 0.034* |

Note: *P<0.05 **P<0.01 ***P<0.001

† Participants can select more than one option for those questions.

**Multimedia Appendix Table 4: The association of technology proficiency and patient perceptions of using patient portals to understand and review lab test results**.

| Patient perceptions | Total (n = 203), n(%) | Technology Proficiency | | | | | *P* value |
| --- | --- | --- | --- | --- | --- | --- | --- |
|  |  | 1: Low  (n=1) | 2: Low-Medium  (n=2) | 3: Medium  (n=52) | 4: Medium-High  (n=81) | 5: High  (n=67) |  |
| **I am comfortable with using patient portals to review my lab results.** |  |  |  |  |  |  | 0.093 |
| Agree | 185 (91.1) | 0(0) | 2(100) | 47(90.38) | 72(88.89) | 64(95.52) |  |
| Neither agree nor disagree | 18 (8.9) | 1(100) | 0(0) | 5(9.62) | 9(11.11) | 3(4.48) |  |
| Disagree | 0 (0.0) | 0(0) | 0(0) | 0(0) | 0(0) | 0(0) |  |
| **I never had any trouble checking my test results on the patient portal?** |  |  |  |  |  |  | 0.002** |
| Agree | 161 (79.3) | 0(0) | 2(100) | 33(63.46) | 67(82.72) | 59(88.06) |  |
| Neither agree nor disagree | 25 (12.3) | 0(0) | 0(0) | 14(26.92) | 9(11.11) | 2(2.99) |  |
| Disagree | 17 (8.4) | 1(100) | 0(0) | 5(9.62) | 5(6.17) | 6(8.96) |  |
| **I find the patient portal can make me review my tests quickly.** |  |  |  |  |  |  | <0.001*** |
| Agree | 187 (91.6) | 0(0) | 2(100) | 43(82.69) | 75(92.59) | 67(100) |  |
| Neither agree nor disagree | 15 (7.4) | 1(100) | 0(0) | 8(15.38) | 6(7.41) | 0(0) |  |
| Disagree | 1 (1.0) | 0(0) | 0(0) | 1(1.92) | 0(0) | 0(0) |  |
| **I find the patient portal is useful to understand my lab results.** |  |  |  |  |  |  | 0.021* |
| Agree | 166 (81.3) | 0(0) | 1(50) | 41(78.85) | 66(81.48) | 58(86.57) |  |
| Neither agree nor disagree | 30 (14.8) | 0(0) | 0(0) | 9(17.31) | 12(14.81) | 9(13.43) |  |
| Disagree | 7 (3.9) | 1(100) | 1(50) | 2(3.85) | 3(3.7) | 0(0) |  |
| **I find the patient portal provides enough useful resources to understand my lab results.** |  |  |  |  |  |  | 0.153 |
| Agree | 143 (70.4) | 0(0) | 1(50) | 34(65.38) | 58(71.6) | 50(74.63) |  |
| Neither agree nor disagree | 35 (17.2) | 1(100) | 0(0) | 13(25) | 14(17.28) | 7(10.45) |  |
| Disagree | 25 (12.4) | 0(0) | 1(50) | 5(9.62) | 9(11.11) | 10(14.93) |  |
| **I have used the resources provided by patient portals to understand my results.** |  |  |  |  |  |  | 0.221 |
| Agree | 131 (64.0) | 0(0) | 0(0) | 36(69.23) | 50(61.73) | 45(67.16) |  |
| Neither agree nor disagree | 40 (19.7) | 1(100) | 1(50) | 10(19.23) | 18(22.22) | 10(14.93) |  |
| Disagree | 32 (16.3) | 0(0) | 1(50) | 6(11.54) | 13(16.05) | 12(17.91) |  |
| **Is there anything that would make the portal better for you?**† |  |  |  |  |  |  |  |
| Make it more user-friendly | 61 (29.1) | 0(0) | 1(50) | 17(32.69) | 25(30.86) | 18(26.87) | 0.817 |
| Allow me to send a message to my physician | 83 (40.0) | 0(0) | 1(50) | 26(50) | 39(48.15) | 17(25.37) | 0.009** |
| Include a health encyclopedia that contains more information about the test | 99 (47.3) | 1(100) | 0(0) | 25(48.08) | 44(54.32) | 29(43.28) | 0.32 |
| Provide timely test result explanation and follow-up instructions | 108 (52.7) | 0(0) | 1(50) | 21(40.38) | 44(54.32) | 42(62.69) | 0.074 |
| Other | 2 (1.0) | 0(0) | 0(0) | 1(1.92) | 0(0) | 1(1.49) | 0.53 |

Note: *P<0.05 **P<0.01 ***P<0.001

† Participants can select more than one option for those questions.
